# Deciphering Nuclear Mechanobiology in Laminopathy

**DOI:** 10.3390/cells8030231

**Published:** 2019-03-11

**Authors:** Jungwon Hah, Dong-Hwee Kim

**Affiliations:** KU-KIST Graduate School of Converging Science and Technology, Korea University, Seoul 02841, Korea; jwhah@korea.ac.kr

**Keywords:** lamin, laminopathy, cytoskeleton, nucleus

## Abstract

Extracellular mechanical stimuli are translated into biochemical signals inside the cell via mechanotransduction. The nucleus plays a critical role in mechanoregulation, which encompasses mechanosensing and mechanotransduction. The nuclear lamina underlying the inner nuclear membrane not only maintains the structural integrity, but also connects the cytoskeleton to the nuclear envelope. Lamin mutations, therefore, dysregulate the nuclear response, resulting in abnormal mechanoregulations, and ultimately, disease progression. Impaired mechanoregulations even induce malfunction in nuclear positioning, cell migration, mechanosensation, as well as differentiation. To know how to overcome laminopathies, we need to understand the mechanisms of laminopathies in a mechanobiological way. Recently, emerging studies have demonstrated the varying defects from lamin mutation in cellular homeostasis within mechanical surroundings. Therefore, this review summarizes recent findings highlighting the role of lamins, the architecture of nuclear lamina, and their disease relevance in the context of nuclear mechanobiology. We will also provide an overview of the differentiation of cellular mechanics in laminopathy.

## 1. Introduction

Mechanical stimuli are the key to controlling numerous biological processes, including proliferation, differentiation, and migration [1,2,3]. Integrin mediates the transduction of forces from the external microenvironment to the intracellular cytoskeleton, and the nucleo-cytoskeletal molecular connections transmit the forces to the intranuclear chromosomal organizations [4,5]. A force-induced nuclear lamina triggers the deformation of intranuclear structures (e.g., chromatin and nuclear bodies) and initiates changes in gene regulation [6,7]. As the center of the mechanical response of a cell, the nucleus properly adapts to mechanical stimuli by altering its position and characteristics during the regulation of cellular functions [8,9].

The nuclear envelope consists of two membranes where nuclear pore complexes (NPCs) are embedded for material exchange [10]. Inside the inner nuclear membrane (INM), A-type and B-type lamins constitute the nuclear lamina, a thin mesh-structured network acting as a viscoelastic shell [11]. The nuclear lamina contributes to the mechanical stress resistance of the nucleus and is essential to maintaining the integrity of the nuclear envelope [12,13]. Lamins are connected to the intranuclear chromatin and cytoskeleton in the lamina [14]. Thus, the nuclear lamina controls the nuclear morphology and mechanics, resulting in epigenetic regulation as well as vital cell behaviors, such as cell polarization and motility [2,15,16,17]. Diseases associated with lamin mutations are typically characterized by nuclear instability, abnormal cytoskeletal architecture, and defective nucleo-cytoskeletal transmission of cellular forces [17,18,19].

*LMNA* mutations induce a complex set of pathological conditions collectively termed laminopathies [20,21,22]. Laminopathies can be induced by mutations in genes related to the nuclear envelope (*LMNA*, *LMNB1*, *LMNB2*, *EMD*, *LAP2*, *LBR*, *ZMPSTE24*, *SYNE-1*, *NUP62*) [23,24]. Laminopathies are mainly associated with tissue-specific defects in load bearing at the nuclear level that may lower the endurance of cells against physical forces. In Hutchinson–Gilford progeria syndrome (HGPS) cells, progerin, a mutant lamin, accumulates in the lamina, which contributes to the thickening of the nuclear envelope [25]. Wild type prelamin A undergoes several post-translational modifications, resulting in mature lamin A lacking the last eighteen amino acids [26]. Mutational errors in prelamin A processing generate progerin and result in the decline of specific cellular functions by increasing genomic instability associated with abnormal epigenetic modification [27,28,29].

Nuclear mechanics are affected not only by nuclear lamin, but also by the cytoskeleton. Nuclear shape is controlled precisely by the perinuclear actin architecture and microtubules [30]. Altered nuclear mechanics observed in laminopathies induce abnormal cellular behavior via damaged nucleus–cytoskeleton connections. From a mechanobiological perspective, this review summarizes the functions of lamin in nuclear mechanics and the evidence underlying molecular mechanisms in laminopathies, focusing on the interaction between mutant lamin and nucleo-cytoskeletal connections.

## 2. Role of Lamins in Nuclear Mechanics

Lamins in the nuclear envelope regulate the mechanical properties and nuclear responses to extracellular stimuli [31]. Since laminopathic cells display nuclear rheology distinct to that of normal cells, aberrant nuclear mechanics is attributed to the development of laminopathies. To further understand the etiology of laminopathies, the role of lamins in nuclear mechanics will be discussed.

### 2.1. Lamin and LINC Complex

**Figure 1:**
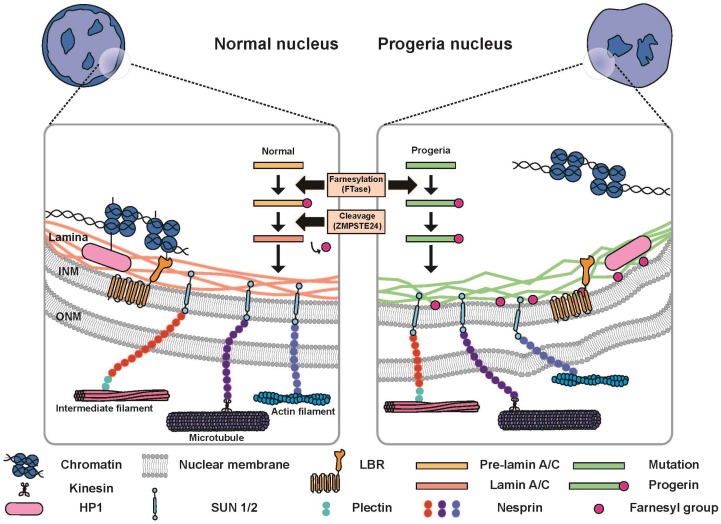
Nuclei of a normal cell and progeria cell. Left: The nuclear lamina is composed of lamin A and C (lamin A/C). Lamin A/C processing consists of multiple steps: (i) The farnesyl group is added to pre-lamin A/C by FTase; and (ii) cleavage by the zinc metalloproteinase Ste24 homologue (*ZMPSTE24*) finalizes the maturation. Heterochromatin is bound to the nuclear envelope by interactions with HP1 and LBR. Right: The nucleus of the progeria cell does not have heterochromatins bound to the nuclear envelope. Cleavage by the zinc metalloproteinase Ste24 homologue (*ZMPSTE24*) is not involved in the process of progerin production. Progerin accumulation in the nuclear lamina contributes to the lamina thickening.

The nuclear envelope is composed of double-layered membranes: the INM and outer nuclear membrane(ONM). The INM surrounds the genome and converges with the ONM to form the nuclear envelope. The gap between the INM and ONM is called the perinuclear space (Figure 1) [10]. The NPC converging INM and ONM mediate the transfer of molecules between the nucleoplasm and cytoplasm. Material exchange through the NPCs mainly depends on the size of the molecules. The import and export of molecules larger than 40 kDa is strictly regulated by nuclear pore complexes; however, molecules with a mass less than 40 kDa or a diameter less than 5 nm diffuse freely across the nuclear membrane [32]. Inner nuclear membrane proteins, e.g., lamin B receptor (LBR), lamina-associated polypeptides (LAPs), emerin, MAN1, and Sad 1p.UNC-84(SUN) domain proteins, interact with proteins facing the nucleoplasm, such as lamins and intranuclear chromatins [33,34]. Mammalian cells consist of two types of lamins: the A-type lamins, including lamins A and C formed by alternative splicing of the *LMNA* gene, and B-type lamins, including lamins B1 and B2 that are expressed by *LMNB1* and *LMNB2*, respectively [35].

Lamin undergoes a series of sequential post-translational modifications via farnesylation, a type of prenylation that modifies conserved cysteine residues at, or near, the C-terminus of proteins by adding lipid intermediates derived from cholesterol synthesis [36]. Farnesylation of lamins A, B1, and B2 is catalyzed by farnesyltransferase (FTase), which facilitates hydrophobic interactions with the INM by adding a farnesyl group to the cysteine in the CAAX motif, while lamin C lacks the CAAX motif (Figure 1) [26,37]. The maturation of lamin A is completed via the endoproteolytic removal of the 15 tail domain residues by a zinc metalloproteinase Ste24 homologue (*ZMPSTE24*), and the mature lamin A is assembled to the nuclear lamina [38,39,40]. For the B-type lamins, the 15 tail domain residues not removed by *ZMPSTE24* are attached to the nuclear envelope [41]. Progerin is created by skipping the last *ZMPSTE24* cleavage step, and is permanently anchored to the INM [26].

Lamin plays an essential role in linking the nucleus and cytoskeleton, and is one of the key components constituting the linker of the nucleoskeleton and cytoskeleton (LINC) complex, which transmits mechanical forces from the cytoskeleton to the nuclear lamina [42]. External forces can be transmitted to the nucleus independent of the LINC complex in specific cases, but not always [43]. “Nucleo-cytoskeleton” is a short form for nucleus–cytoskeletal interaction [44]. Nuclear components that interact with the cytoskeleton are SUN proteins, nesprin, and the nucleoskeleton. The nucleoskeleton, which is formed by networks of lamin, as well as lamin-binding proteins, is mainly located inside, and near to, the nuclear envelope [45]. Nuclear chromatin and chromosomes interact with lamin, like most inner nuclear membrane proteins that contribute to nuclear architecture [45]. The LINC complex is comprised of nesprins containing SUN (Sad1 and UNC-84) and a C-terminal KASH (Klarsicht, ANC-1, and Syne homology) domain (Figure 1) [46]. Many SUN domain proteins interact with lamins and are localized to the nuclear envelope by functional lamin [47,48]. The SUN domain proteins are bound to the lamina, chromatin, and NPC [49]. Nesprins connect the nuclear envelope and extranuclear cytoskeleton, where nesprin-1 and nesprin-2 bind to actin and microtubule-associated kinesin and dynein [50]; nesprin-3 interacts with the intermediate filament system [51], and nesprin-4 connects kinesin-1, a motor protein of the microtubule [52]. In this section, we discuss the production process of lamin and the LINC complex that lamin interacts with.

### 2.2. Nuclear Mechanics

Among the diverse group of structural components, such as nuclear lamina, chromatin organization, and cytoskeleton, the nuclear lamina is the major contributor to nuclear mechanical homeostasis. The ability to endure local forces on the nuclear surface is supported by lamin as the primary protein of the nuclear lamina [11,53]. The lamina is the major load-bearing part that provides nuclear stability under tensile stress [54]. A- and B-type lamins are the major components of the nuclear lamina, underlying the distinct rheology of the nucleus [55,56]. Rheology concerns the flow properties of materials, such as colloidal material and biomaterials with viscoelasticity, and is important for understanding the complex characteristics of a cellular system. Recent studies have shown that A-type lamins modulate nuclear viscosity, while the elastic features are mediated by B-type lamins [2,31,57,58]. Lamin A predominantly regulates the mechanical response of the nucleus [57]. Studies have shown that the differences in lamin A expression correlate with tissue stiffness, and bone and muscle tissues with a higher expression of A-type lamin contain stiffer nuclei than brain or adipose cells, while B-type lamin is constitutively expressed in all types of cells [2,59]. Moreover, nuclear stiffness is known to be determined by the differential expression between A- and B-type lamins, where the expression of A-type lamin is critical to nuclear integrity, as lower levels of A-type lamin increase the fragility and risk of deformation of the nucleus.

It is crucial to maintain nuclear shape regardless of mechanical stress because an abnormal nuclear shape contributes to pathological outcomes [60,61,62]. Nuclear shape is altered by the nucleo-cytoskeletal structure and connections in response to extracellular physical stimuli. Increased expression of A-type lamins enhances nuclear stiffness and prevents deformation. The migration of cells during cancer metastasis and leukocyte extravasation dynamically alters the nuclear morphology following deformation in cell shape [63,64]. Morphological fluctuations in the cell, in turn, influence the nuclear morphology at the microscale. For example, elongated cells and nuclei occur in stripe-shaped fibronectin-coated surfaces, and a round morphology is typically observed in round-shaped fibronectin-coated surfaces, which is mediated by the formation of a perinuclear actin cap structure containing nesprin and actin filaments. Depending on the shape of the cells, myosin 2 activation and actin formation, which are regulated by Arp2/3 activation, contribute to nuclear morphology [65]. The nuclear shape is controlled by F-actin in the dome-like actin cap, which is connected to the nucleus by nesprin, SUN, and lamina. Treatment with blebbistatin and/or trypsin inhibits actomyosin contractility and causes actin disorganization, and ruins the ability of the actin cap to regulate the nuclear shape. Therefore, treatment with blebbistatin or trypsin induces the formation of round-shaped nuclei, which suggests inadequate actin synthesis in the cytoplasm [16,66,67,68]. Regarding the adhesive stripe-micropatterned substrate, mouse embryonic fibroblasts lacking A-type lamins were used as a model of dilated cardiomyopathy and muscular dystrophy, and mouse adult fibroblasts from *LMNA*^L530P/L530P^ mice served as a model of progeria, which failed to form an actin cap and control the nuclear shape [69].

Though lamin A predominantly contributes to nuclear mechanics, other nuclear components account for the nuclear mechanical response [2,31]. Chromatin, one of the regulators of nuclear mechanics, controls the nuclear response to external force [54,70,71]. Chromatin in the interphase nucleus is mechanically regulated by modulating chromatin condensation [57]. Therefore, cell compression leads to nuclear stiffness with increasing chromatin condensation and altered transcriptional responses determined by cellular geometry [72]. Changes in chromatin condensation, via the regulation of methylation, acetylation, cell compression, and osmotic pressure, control the mechanical properties of the nucleus. Since hyperacetylation can be induced by micropipette aspiration, heterochromatin formation decreases, and nuclear softening can be induced [57]. Mechanically pushing cells downward onto the plate increased chromatin condensation [72]. This compression reduced actomyosin contractility and upregulated the nuclear translocation of HDAC3, resulting in increased heterochromatin synthesis and decreased euchromatin levels. Intranuclear mechanics and transcription mechanisms can be modulated via control of chromatin condensation by exerting force on the nucleus [72]. As mentioned above, chromatin controls the nuclear properties, and lamin A mutations decrease the heterochromatin content and chromatin tethering to the nuclear envelope [27,73]. As a result, lamin mutants exhibit altered nuclear stiffness and viscosity [73]. Nuclear mechanics can be controlled by a combination of lamin, chromatin, and cytoskeleton near the nucleus.

### 2.3. Nuclear Response to External Mechanical Force

Several experiments, including those involving microfluidic chips, ballistic particle injection and tracking, micropipette aspiration, atomic force microscopy (AFM), and compression with microplates, have demonstrated the elastic and viscous characteristics of the nucleus [74]. More importantly, rheological analysis of cell nuclei has revealed that the nuclear response is dependent on the structure of the lamina and intranuclear components, since lamin interacts with nuclear envelope proteins, chromatin, and transcription factors. These interactions control the stiffness of the nucleus based on transcription, and nucleoskeletal or chromatin structure. Laminopathic cells display altered size, shape, dynamics, and nucleus stiffness.

To characterize the changes in the axial and transverse dimensions of the nucleus and cell before and after compression, microchannels were designed for the high-throughput rheological analysis of cells [75]. With this chip, the deformation and recovery of nuclear size can be demonstrated before and after compression. Microscopic imaging and quantitative analysis of embryonic stem cells showed nuclear responses to mechanical stress along the major axis, as well as size and deformability of the nucleus during entry into the small microchannel. Based on these results, the authors observed an auxetic nuclear interior, which was thicker perpendicular to the axis of the applied strain [75].

High-throughput ballistic injection was also used to measure the movement of microspheres to identify the viscoelastic properties of the intranuclear compartments [76,77]. Fluorescent microspheres were injected into the nuclei [77] and the mechanical responses of the nuclei to shear stress were assessed by tracking the trajectory of fluorescent particles and calculating the mean squared displacement (MSD) [77]. The MSD of fluorescent particles inside the nucleus and cytoplasm suggests a slower motion of the nuclear interior than that of the cytoplasm, indicating a higher elasticity and stiffness of the nucleus when compared with the cytoplasm [77].

Aspiration of the nucleus into a micropipette with a diameter of 3–6 µm and microscopic monitoring of nucleus was utilized to estimate the plasticity of the nucleus [70,78]. Microscopic aspiration of swollen and unswollen nuclei revealed no differences in nuclear response to external force. However, chromatin was detached from the nuclear lamina in swollen nuclei, which suggested that the nuclear lamina mainly determines the mechanical property of the nucleus. Factors in the nucleoplasm control nuclear deformation after severe extension [79]. HGPS nuclei were slightly more resistant to aspiration than healthy nuclei [25]. However, the HGPS lamina was less deformable than the control nuclei after aspiration for 5 min, while the stiffness of control and HGPS lamina were similar after aspiration for 30 s, indicating that HGPS cells failed to adapt to external mechanical stress. Therefore, failure in mechanotransduction induces fragility in the HGPS nucleus, especially in load-bearing tissues, which is related to the mechanism of laminopathies [80].

AFM measures the applied force and depth of indentation while applying a highly localized force to directly induce nuclear deformation [78]. The AFM measurement showed that the Young’s modulus of a 61-year-old healthy donor and a HGPS patient was much higher than that of a 4-year-old healthy donor, suggesting that the nuclear stiffness of HGPS cells was similar to that of cells derived from an old donor, since progerin accumulates with time, as seen during natural aging [81].

Intranuclear responses under shear stress and compression were observed using particle tracking of fluorescent-tagged upstream binding factor one (UBF1-GFP) and fibrillarin (Fib-GFP) [82]. UBF1 is involved in decondensation and is located in ribosomal gene repeats [83]. UBF1 and fibrillarin are nucleolar markers, mediating both chromatin and intranuclear protein dynamics [82]. Under cellular shear stress, the direction of intranuclear components followed the direction of flow and further compression dynamically modulated intranuclear movement [82]. These results suggest that nuclear mechanics in response to applied forces might alter the cellular phenotype [82]. So far, we have addressed the rheological properties of the nucleus according to how the nucleus reacts to mechanical stimuli. The next section deals with changes in the rheological properties of the nucleus by the altered interaction between the nucleus and cytoskeleton in laminopathic cells.

## 3. Nucleus–Cytoskeleton Connections in Laminopathy

Nuclear lamins mediate the interaction between the nucleus and cytoskeleton by modulating nuclear mechanics, nuclear morphology and positioning, cell migration, mechanosensation, apoptosis, and differentiation. Mechanical imbalance between the nucleus and cytoskeleton can ruin cellular homeostasis. Cellular dysfunction induced by defects in lamin production leads to the development of diseases involving load-bearing tissues, such as muscle and bone disorders, e.g., Emery–Dreifuss muscular dystrophy (EDMD), dilated cardiomyopathy (DCM), and osteoporosis [19,84,85,86]. Furthermore, neuropathy, accelerated aging, dermopathy, and lipodystrophy have been identified as correlating with the nucleus–cytoskeleton connections mediated by lamins. The mechanobiological aspects of abnormal nucleus–cytoskeleton interaction, inducing disruption of cellular function in laminopathies, will be presented.

### 3.1. Reduced Mechanical Stability of Nucleus in Laminopathy

Laminopathic cells contain nuclei with reduced mechanical stability due to the altered composition of the lamina [87]. Nuclei derived from EDMD and HGPS also display an altered nuclear shape and structure, an abundance of lobulations, thicker lamina, and heterochromatin that have detached from the nuclear envelope (Figure 1) [88,89,90]. As lamin is essential to the maintenance of nuclear mechanical stability, abnormal lamin A and C (lamin A/C) production increases sensitivity to mechanical stress and interrupts mechanotransduction signaling [42,91]. Cells derived from EDMD mice carry deformed nuclei, which show decreased cytoskeletal stiffness and dysregulation of genes involved in mechanosensing of the cellular environment [92]. The nuclei of fibroblasts, cardiac, and skeletal muscle cells derived from laminopathic patients are susceptible to mechanical stress and are easily disrupted [19,93,94]. HGPS cells contain stiffer nuclei than that of normal cells [25,91]. These traits accumulate and become more severe with increasing passage number [88]. Despite elevated nuclear stiffness in HGPS cells, the sensitivity to mechanical stress is high, and therefore, smooth muscle cells associated with the blood vessel are reduced [91]. Since lamin recruits SUN-KASH proteins to the nuclear envelope, nucleus–cytoskeleton connections are suppressed in laminopathic cells. The mechanical stress induces diseases associated with load-bearing tissues, which exhibit defects involving nucleus–cytoskeleton connections, mechanical signal transduction pathways, cell differentiation, gene regulation, and proliferation [80]. EDMD mice exhibit muscular dystrophy and dilated cardiomyopathy, and even die before the age of eight weeks [85]. Altered nuclear fragility in laminopathies reinforces the role of the lamin network in ensuring nuclear mechanical stability in normal cells. A significant feature of laminopathies is nuclear rupture and repair by the disruption of nuclear lamina. A large gap in the lamina, where there is a lack of low INM proteins, such as B-type lamins, NPCs, and inner nuclear membrane proteins, has been observed in laminopathic cells [95]. Chromatin herniation from weak membrane integrity has also been observed in cells with mutations in lamin and lamin-associated proteins [85,96]. Transient nuclear rupture induces reduced nuclear integrity and the temporal exchange of cytoplasm and nucleoplasm [97]. Apoptosis and division normally happen in cells with nuclear rupture, suggesting that nuclear rupture is not fatal to the cell. However, repetitive nuclear rupture causes permanent translocation of protein complexes, such as PML bodies, myeloid bodies, vesicles, and mitochondria [93,95]. Therefore, nuclear rupture is one of the major contributors to laminopathies.

Diseases can be managed based on our understanding that lamins play an important role in maintaining the nuclear mechanical stability [98]. HGPS is one of the representatives in laminopathies. In HGPS cells, progerin, a permanently farnesylated protein, accumulates in the nuclear envelope. Farnesyltransferase inhibitors (FTI) are suggested for the treatment of HGPS, as they decrease the accumulation of progerin in the nuclear envelope [99,100]. FTI treatment increased wound healing, persistent migration, and restored nuclear stiffness; however, mechanical sensitivity failed to improve [91]. Despite the improved nuclear phenotypes of HGPS following FTI treatment, the outcome was not perfect or complete [98]. Based on these results, permanent farnesylation of progerin is not the only way to transform disease phenotypes.

Though B-type lamin is not the main contributor to nuclear mechanics when compared to A-type lamin, lamin B1 can cause an irregular nuclear shape or nuclear blebbing [80]. Lamin B1-deficient nuclei spin around within the cell, suggesting that lamin B1-deficient cells have abnormal nuclear anchoring [101]. In addition, lamin B1-deficient cells struggle to proliferate, while lamin B2-deficient cells are normal in cell proliferation [102]. For example, lamin B1-deficient cells show defects in chromosome positioning, polyploidy, slow growth, and accelerated senescence [103]. Lamin-mutated cells have reduced nuclear integrity. Defects in nuclear integrity induce susceptibility to mechanical stress in lamin-mutated cells. This vulnerability can be recovered by correcting the impaired lamin processes.

### 3.2. Nucleus–Cytoskeleton Connections in Laminopathy

The nucleus interacts with intracellular structures in response to mechanical signals [5]. LINC complexes, which mediate extracellular mechanical signals, interact with the cytoskeleton. Uncoupling of nucleus–cytoskeleton connections is critical to ensuring the integrity of the nuclear envelope in laminopathies [104]. Since lamin recruits SUN-KASH proteins to the nuclear envelope, nucleus–cytoskeleton connections are suppressed by the loss of localization of SUN-KASH proteins in lamin knockout cells. SUN1 in lamin knockout cells is overexpressed in the nuclear envelope and Golgi apparatus, which promotes nuclear herniations and cellular toxicity [105]. We will now discuss how the nucleus–cytoskeleton connections affect the nucleus, and the role of lamin mutations in the inhibition of this interaction depending on the type of cytoskeleton.

Actin stress fibers located near the nucleus are connected to the nuclear envelope via LINC proteins, including lamin A/C [69,106,107]. Actin fibers induce internal tension and morphological changes of the nucleus. The presence of irregularly shaped nuclei with few organized links between lamin and the cytoskeleton has been correlated with progeria and Alzheimer’s disease [108,109]. Nuclear lamin A/C and intranuclear structure have been shown to be redistributed along the basal–top axis via actin capping [110]. In addition, cyclic stretching induced nuclear flattening by pushing the nucleus, while formation of an actin cap reduced the nuclear height and led to the conservation of the nuclear 2D-projected shape during cyclic mechanical stress [111]. In *LMNA*^−/−^*LMNB1*^−/−^*LMNB2*^−/−^ cells (triple knockout, or (TKO) cells), the nuclei showed an irregular shape and were easily ruptured [112]. DNA damage was more frequently observed in TKO cells than in normal cells [112]. When cortical actin was eliminated with cytochalasin D, the nuclei in TKO cells—which ordinarily do not have actin caps—did not experience any mechanical stress [112]. Therefore, the nuclei in cytochalasin D-treated TKO cells were resistant to rupture [112]. When cells adhere to the substrate, the nuclei maintain a flattened round shape. During detachment, the volume decreased by about 50 percent, whereas the nuclear volume is increased by the inhibition of myosin phosphorylation and the polymerization of F-actin and microtubules [113]. These findings suggest that the nucleo-cytoskeleton also controls the nuclear volume by compressing the nucleus [114,115,116].

Nuclear ruptures and DNA damage are aggravated by mechanical stretching, and microtubules affect fluctuation in the nuclear membrane. Microtubules bound to the nuclear envelope play a crucial role in the breakdown of the mitotic nuclear envelope and nuclear lobulations [117]. Lamin A and microtubules are correlated with the maintenance of nuclear morphology, [30] which regulates molecular translocation between the nucleus and the cytoplasm, affecting gene regulation [118].

Lamin A/C deficiencies induce the detachment of desmin intermediate filaments from the nuclear surface. Desmin is a muscle-specific intermediate filament that connects not only the nucleus, but also myocytes [119]. This connection, mediated via plectin 1, is essential for muscle integrity [120,121]. Defects in desmin result in the release of nuclear tension and changes in mechanoresponsive gene expression [121]. Lamin mutations disrupt the desmin network, which triggers nuclear deformation [85] and contractile dysfunction [122]. Studies based on ballistic intracellular nanorheology showed significant changes in the cytoskeletal stiffness of wild-type and *LMNA* mutant cells [123]. Deficiency of lamin A on the nuclear envelope decreased the cytoskeletal stiffness [80]. The relationship between lamin A and cytoskeletal stiffness suggests that direct physical interaction between the nucleus and cytoskeleton can also be changed via alterations in the nuclear envelope. Finally, defects in *LMNA* exacerbated a variety of cardiopathies and muscular dystrophy [122]. In physiological aspects, decreased cellular stiffness of *LMNA* cells suggests that the molecular mechanism of laminopathies is vulnerable in muscle cells, which are readily exposed to mechanical stress [123].

### 3.3. Chromatin Organization and Gene Regulation in Laminopathy

To understand the cellular mechanoresponses, an appreciation of the mechanism of how external mechanical signals are transmitted to the nucleus and how the signals alter the genome [124] is required. Chromatin movement via transduction of mechanical signaling along cytoskeleton–lamina molecular connections is crucial in gene regulation [125]. Eukaryotic cells contain organelles and chromatin, which is orderly compacted with histone and non-histone proteins within the nucleus at the interphase [126]. The organization of chromatin affects nuclear architecture and cellular function by regulating the process of DNA compaction and the condensation of DNA [127]. Heterochromatin is distinguished from euchromatin by a different level of DNA condensation at interphase [128]. Euchromatin is less condensed and more easily transcribed, while heterochromatin has a much more condensed and highly ordered structure [129]. Heterochromatin is mainly located near the nuclear envelope, while euchromatin is mainly located in the internal nucleus. By changing the condensation of DNA, the access of transcriptional factors is regulated [128,130]. Mechanoreactive lamin interacts with DNA directly, or indirectly via complexation with LEM domain proteins [131]. Lamin disorganization interferes with RNA polymerase II activity, suggesting that lamin serves as a scaffold for transcription complexes used in gene regulation, with increased activity of RNA polymerase II [132].

Actin and microtubules affect lamin integrity and even nuclear chromatin dynamics [73]. Mechanical signals alter nuclear dynamics by modulating actomyosin forces mediated by lamin A/C and the LINC complex [133]. Myosin contractility in the cytoskeleton and the integrity of lamin A/C synergistically control chromatin dynamics in response to mechanical cues [134]. Static external compression of the nucleus triggers the upregulation of HDAC3 shuttling and heterochromatin content via reduced actomyosin contractility [72]. As chromatin organization is mediated by interaction between the nucleus and cytoskeleton, chromatin structure is disorganized in laminopathic cells, leading to impaired adipogenesis [135].

Nuclear translocation of transcription factors, e.g., Yes-associated protein (YAP), nuclear factor-κB and Rel (NF-κB), and myocardin-Related Transcription Factor (MRTF), is regarded as an important regulator of gene expression in response to physical cues. Activation of these factors is mediated by actomyosin contractility and upregulation of laminopathic cells [80,136,137,138,139,140,141,142,143]. YAP/TAZ activity is regulated by mechanical cues, which are mediated by F-actin [139]. Compared to normal cells, *LMNA* mutant myoblasts display increased YAP signaling, which disrupts mechanosensing ability [140]. NF-κB activity is also regulated by actomyosin contractility. Activation of alpha6/beta4 integrin of focal adhesions facilitates Rac1 and Cdc42, resulting in the activation of NF-κB [141]. Although NF-κB binding to the transcription factor was enhanced in *LMNA*^−/−^ cells compared to WT cells, NF-κB-regulated activity was decreased in the *LMNA*^−/−^ cells, indicating the role of lamin A/C in transcription activity [80]. A model of Duchenne muscle dystrophy (DMD) also showed upregulation of NF-κB, which was enhanced by mechanical stretch [142]. Lamin mutation or loss induces emerin dysregulation, resulting in the impaired nuclear localization of mechanosensitive transcription co-factor, myocardin-related transcription factor A (MRTF-A). MRTF-A is related to cardiac function because emerin and lamin control gene expression that regulates actin polymerization and actin dynamics, affecting MRTF-A location [143]. These findings suggest that lamin mutations play a role in laminopathies by dysregulating gene expression. Lamin mutations induce the pathological properties of laminopathies by dysregulation in the organization of chromatin and interactions with transcription factors.

## 4. Cell Functions Related to Nuclear Mechanics in Laminopathy

Nuclear mechanics and nucleus–cytoskeleton coupling play a role in cellular function [144]. Altered nuclear mechanics mediated by mutations in lamins and LINC complex proteins induce aberrant cellular functions. Here, we discuss how cellular functions are related to the nucleo-cytoskeleton in laminopathies.

### 4.1. Nuclear Positioning

**Figure 2:**
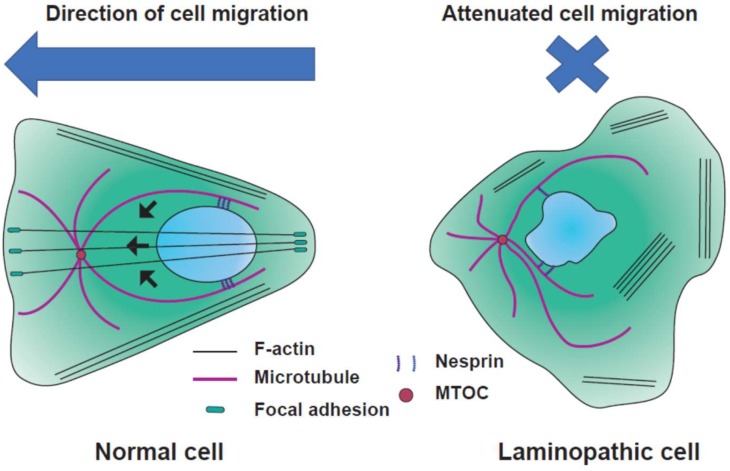
Nuclear positioning in laminopathic cell migration. Left: Normal cells are polarized in the order of “front-end nucleus– microtubule-organizing center (MTOC) rear end”. Black arrows indicate the direction of nuclear migration. Blue arrow shows the direction of cell migration. Right: Laminopathic cells have a deformed nucleus and weak connection between the nucleus and cytoskeleton. Since F-actin formation is disturbed in laminopathic cells, laminopathic cells lose their directionality in cell migration.

Nuclear positioning, which involves well-controlled movements by elements of the cytoskeleton and protein complexes in the nuclear envelope, is required for cellular migration and differentiation (Figure 2) [23,145]. Lamin functions as a scaffold, enabling the cytoskeleton to localize at the INM, which implies that loss of lamin diminishes nuclear positioning. Microtubule motors transfer intracellular cargo, and the nucleus is the biggest cargo for kinesin and dynein. While nuclear positioning, which is mediated by nucleus–microtubule-organizing center (MTOC) connections, is essential for neural development [146], nuclear positioning is accentuated by microtubules growing toward the dorsal–ventral (DV) axis [116]. In laminopathic models, the nucleus-MTOC connection is inhibited, resulting in impaired nuclear positioning [123]. Actin-mediated nuclear positioning is effective in autosomal dominant Emery–Dreifuss muscular dystrophy (AD-EDMD) [147]. Nuclear dislocation induces the loss of neuromuscular junction in AD-EDMD [148]. Cells obtained from synaptic muscle fibers of EDMD mice have nuclei which are dislocated at neuromuscular junctions [148]. Emerin, encoded by the EMD gene, is one of the best described lamin-interacting proteins, and *LMNA* are responsible for AD-EDMD [21,149]. Lamin-null fibroblasts and emerin-null fibroblasts exhibit defective nuclear positioning and nucleus–cytoskeletal connections [150].

Emerin interacts with myosin IIB to polarize the transmembrane actin-associated nuclear (TAN) line, driving nuclear translocation [150]. At the surface of the nucleus, emerin, SUN, and KASH proteins are aligned with F-actin, leading to the formation of transmembrane actin-associated nuclear (TAN) lines. TAN lines promote nuclear movement via retrograde actin flow. A-type lamin is needed to couple TAN lines to the nucleus and centrosome orientation [147,151]. In migrating neurons, activated myosin II, which is at the rear edge, can break adhesions at the trailing edge and contract to move the nucleus forward (Figure 2) [152]. Emerin-null mice showed no specific dystrophic phenotype except reduced muscle regeneration, suggesting that lamin A/C might be the main factor underlying muscle function, and emerin enhanced lamin A/C function [153,154]. Finally, in lamin-defective cells, defective connections between the nucleus and cytoskeleton, actin, and MTOC lead to nuclear mispositioning.

### 4.2. Cell Migration

The organization of lamin A and the cytoskeleton contributes to altered cell migration [155]. During cell migration, the actin cap controls nuclear polarization and migration (Figure 2) [107]. Migrating cells display an elongated nucleus compared with round nuclei in non-migrating cells [107]. Therefore, the direction of actin cap formation predicts the direction of cell migration [107]. Nucleus–cytoskeleton coupling is essential for 3D cell migration [156]. The nucleus is stiffer than the cytoplasm, which governs the overall cellular behavior during cell migration through narrow channels and deformations [157,158,159]. When cells penetrate through a confined channel, the nucleus changes its shape to adapt to external physical stresses [16]. Poor nuclear expression of lamin A/C contributes to nuclear compliance and easily changes the nuclear shape. However, extremely low levels of lamin A/C expression can easily damage the nucleus [16]. Therefore, moderate expression of lamin A/C is required to ensure nuclear survival during cell entry through small pores [16].

Disruption of the LINC complex attenuated cell adhesion and migration in laminopathic mouse models [123]. Defects in cell migration were mediated by the disorganization of F-actin induced by the loss of binding to lamin A/C in lamin A/C-mutated fibroblasts [160]. *LMNA* knockdown cells showed a diminished expression of cytoskeletal proteins. Although the size of focal adhesions located at the end of the actin filament decreased in *LMNA*-depleted cells, the contractility of actin stress fibers in these cells increased, and thus, exhibited a loss of directionality during cell migration. These results suggest that laminopathic cells exhibit an imbalance between focal adhesions and cytoskeletal contractility due to the loss of A-type lamin [161].

In contrast to A-type lamins, B-type lamins are rarely associated with diseases, except lissencephaly [162]. *LMNB2*-deficient mice have an abnormal layer of neurons in the cerebral cortex and cerebellum. This abnormality is from impaired neuronal migration and defects in nuclear positioning. The early stage of brain development is normal, but after cortical neurons start migration, impairment appears. As neuron progenitors differentiate, they migrate from the ventricular zone to generate the cortical plate [163]. Laminopathic cells show a reduced migration ability because of the disorganized actin structure, changed level of lamin expression, and imbalance of tension between the focal adhesion and actin fibers connected to the nucleus.

### 4.3. Mechanosensation

**Figure 3:**
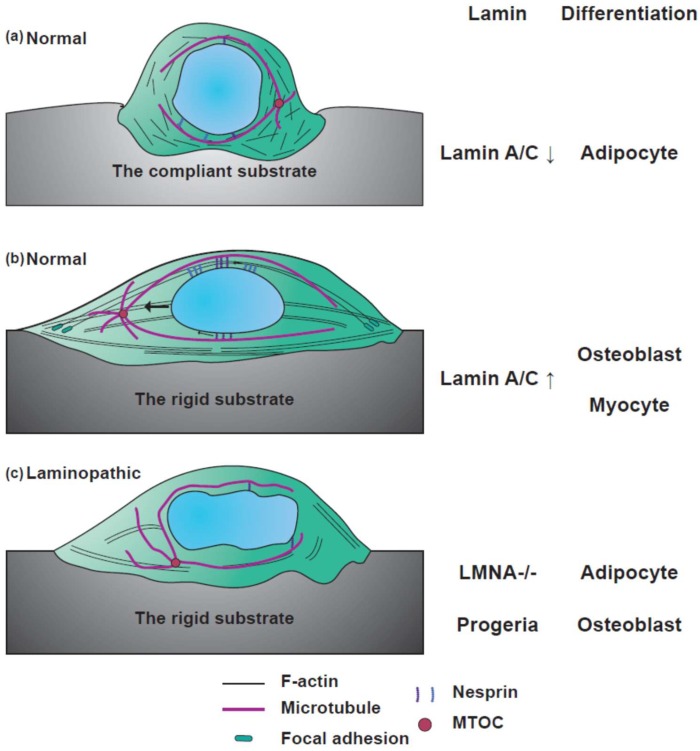
Mechanosensation and differentiation determined by lamin and the substrate stiffness. Cells on hydrogels of different stiffnesses show distinct mechanosensation. Substrate stiffness-dependent stem cell differentiation depends on different lamin A/C expression. (**a**) A cell on the compliant substrate is less spread out, and cytoskeleton formation and nuclear shape maintenance are inhibited by the compliant substrate. On the compliant substrate, lamin A/C expression decreases and the stem cell can differentiate into an adipocyte. (**b**) A normal cell on the rigid substrate is fully spread out with well-organized F-actin and microtubules. Focal adhesions on the rigid substrate are also bigger than on the soft substrate. Lamin A/C expression increases as the substrate stiffness increases. Therefore, stem cells with a rigid nucleus can differentiate into osteoblasts and myocytes. (**c**) Laminopathic cells featuring fragile nuclei show miniscule focal adhesions, even when laminopathic cells are on the rigid substrate. Laminopathic cells display attenuated mechanotransduction due to weak nucleus–cytoskeletal connections.

Cells interact with the microenvironment, which is crucial for various cell functions [144]. Impaired force transmission between the extracellular matrix and intracellular organelles is the most common cause of progression of laminopathic diseases [164]. Lamin is associated with the process of mechanosensation [87,165], which affects cell fate during differentiation and adaptation to mechanical stress (Figure 3) [144]. Lamin A/C-defective cells feature a reduced size of focal adhesions, and an imbalance between focal adhesion formation and cytoskeletal tension, which ultimately hinders the mechanosensing ability [161]. Mechanical tension between the lamina and cytoskeleton is altered by the matrix elasticity. As the matrix rigidity increases, non-muscle myosin II (NMM II)-based contractility and focal adhesion size increases [166]. When myosin is activated and the nucleus is exposed to higher tension, the phosphorylation of lamin is rapidly inhibited following cytoskeletal tension [16]. Defective mechanosensing responses in *LMNA*-mutated cells represent the pathological mechanism underlying laminopathies involving diseases of load-bearing tissues.

Nucleus–cytoskeleton connections enable force transmission from the focal adhesion to the nucleus, which controls the level of lamin expression and the mechanical properties of the nucleus, affecting cell fate [2,43]. Since the physical properties of the microenvironment also modulate nuclear morphology [167], stiff substrates lead to the flattening of the nucleus, while a round nuclear shape features on soft substrates [167]. The round nuclear shape on soft substrates is attributed to low actomyosin contractility, in contrast to the flattened nucleus on stiff substrates due to high actomyosin contractility [167].

EDMD cells with a fragile nucleus and softer cytoskeleton showed poor viability to mechanical stress and exhibited impaired expression of mechanosensitive genes *EGR-1* and *IEX-1*, especially under repetitive strain [80]. Mutations involving lamins disrupt mechanical coupling between the extracellular matrix and nucleus and inhibit mechanotransduction pathways [80].

Changes in nuclear mechanical properties by physical cues around the cell regulate nuclear morphology and permeability of transcription factors [2]. Cells sense tension induced by the extracellular matrix, and altered lamin A phosphorylation and nuclear distribution via the mechanosignaling of Yes-associated protein (YAP) and retinoic acid receptor (RAR) pathways, in an effort to balance cytoskeletal homeostasis [2]. Altered YAP activity has been found in the mechanosensing response of lamin-mutant myoblasts [140]. The degree of F-actin polymerization is one of the regulators of YAP activity [139]. The expression of lamin and nesprin-1 modulates FHOD1-dependent F-actin accumulation, contributing to YAP activation and resulting in the abnormal mechanosensation of lamin and nesprin-1 mutant cells plated on substrates with different stiffnesses [168]. Tension between the lamina and cytoskeleton is dependent on the stiffness of the substrate and cannot guide proper nuclear responses by abnormal lamins, inducing a loss of nucleo-cytoskeletal connections.

### 4.4. Differentiation

Stem cells respond to mechanical cues from the microenvironment and converge those signals to define their lineage [166]. Stem cells exhibit the potential for differentiation to any lineage and the activation of diverse gene expression [169]. Stem cells can change their fate under epigenetic regulation of the chromatin structure, DNA methylation, microRNAs, and transcription factors by mechanical conditions [170,171]. One of the typical features exhibited by stem cells is the lack of lamin A/C, which plays a crucial role in stem cell differentiation. The expression of lamin A increases with the stage of differentiation, and mediates transcriptional processes in differentiation [172,173,174]. Furthermore, the level of lamin expression modulates the lineage of stem cells. Lamin A/C siRNA treatment of MSC inhibited the differentiation of osteoblasts and enhanced the differentiation to adipocytes, while progerin mutants promoted osteogenic differentiation (Figure 3) [175,176]. Substrate stiffness-dependent lamin A/C expression also controls the cell fate [16]. As lamin A is a regulator of nuclear stiffness, stem cells carry softer nuclei [31]. The stiffness of lamin A knockdown cells is similar to that of the stem cells [70]. The nuclei of stem cells are six-fold more deformable than those of differentiated cells [70]. Furthermore, stem cells exhibit higher physical plasticity than differentiated cells. Studies increasingly suggest that stem cell fate is changed by alterations in chromatin structure [70]. As cells differentiate, chromatin becomes condensed, while undifferentiated cells contain diffuse chromatin [171]. Chromatin condensation and lamin A/C regulate nuclear plasticity during stem cell differentiation [70]. Nuclear stiffness can be both a mediator and a result of differentiation. The nucleus in undifferentiated cells is more susceptible to mechanical strain than in differentiated cells. Using mechanosignaling of the Yes-associated protein (YAP), serum response factor (SRF), and retinoic acid receptor (RAR) pathways, cells sense tension in stiff substrates and alter lamin A phosphorylation and distribution in the nucleus, resulting in homeostasis of the cytoskeleton [2]. Tension mediated via actin fibers in cells cultured on stiff substrates induces the dephosphorylation of lamin A and anchoring to the LINC complex, which promotes osteogenic differentiation. Upregulated tension can also activate the SRF pathway associated with myosin IIA, which is known as the feedback-based gene circuit and requires intact nucleo-cytoskeletal connections [16,87]. Lamin mutations inhibit mechanosensitivity to physical surroundings due to abnormal nuclear stiffness and a malfunction in mechanosignaling.

## 5. Conclusions

Mechanical signals alter nuclear mechanics. In cases of laminopathy, due to mutations in lamin genes, the role of the laminopathic nucleus–cytoskeleton differs from that of the normal nucleus. Dysregulation of the nucleus–cytoskeleton induces defects in cellular functions, such as nuclear positioning, cell migration, mechanosensation, and differentiation. Lamin is the core tissue-specific protein of lamina, and therefore mediates mechanotransduction. Laminopathies are mainly observed in load-bearing tissues, such as muscle and bone. Therapeutic approaches targeting the relationship between laminopathy and mechanobiology are promising solutions. Molecular therapy by RNA interference (RNAi) destroys mutant *LMNA* mRNAs selectively and reduces the accumulation of progerin, inducing pathological consequences. RNAi therapy abates mechanically sensitive smooth muscle cells in the HGPS mouse model and improves vascular complications [177]. An impaired autophagy is one of the reasons for cardiomyopathy [178]. Rapamycin treatment promotes autophagy by improving the cellular phenotype, suggesting that rapamycin and its analog are also potential new treatments for laminopathies [179]. Investigation of the molecular mechanisms underlying laminopathies, for example, HGPS or muscle dystrophy, can be used to combat diseases related to aging and muscle, based on a better understanding and insight into the mechanobiology of laminopathy.
